# Why are most colorectal cancers diagnosed outside of screening? A retrospective analysis of data from the English bowel screening programme

**DOI:** 10.1177/09691413221100969

**Published:** 2022-05-16

**Authors:** Robert Stephen Kerrison, Andrew Prentice, Sarah Marshall, Christian von Wagner

**Affiliations:** 1 School of Health Sciences, University of Surrey, Guildford, UK; 2 St Mark’s Bowel Cancer Screening Centre, St Mark’s Hospital, Harrow, UK; 3 Research Department of Behavioural Science and Health, 4919 University College London, London, UK

**Keywords:** Colorectal cancer, screening, inequalities, epidemiology, stage at diagnosis

## Abstract

**Objective:**

Despite several interventions to increase participation in England, most colorectal cancers (CRCs) are diagnosed outside of the screening programme. The aims of this study were to better understand why most CRCs are diagnosed externally, the extent to which this is due to suboptimal uptake of screening, and the extent to which it is due to other factors, such as false-negative test results.

**Setting / Methods:**

We performed a clinical audit of 1011 patients diagnosed with CRC at St Mark's Hospital (Harrow, UK) between January 2017 and December 2020. Data on the diagnostic pathway and screening history of individuals were extracted from the bowel cancer screening system and assessed using descriptive statistics.

**Results:**

446/1011 (44.1%) patients diagnosed with CRC were eligible for screening at the time of diagnosis. Of these, only 115/446 (25.8%) were diagnosed through screening. Among those diagnosed via non-screening pathways, 210/331 (63.4%) had never taken part in screening, 31/331 (9.4%) had taken part but were not up to date, and 89/331 (26.9%) had taken part and were up-to-date (of these, 82/89 [92.2%] had received a normal or weak positive test result, and 5/89 [5.6%] had received a positive result and declined colonoscopy).

**Conclusion:**

Nearly two-thirds of screening eligible patients diagnosed through a non-screening pathway had never taken part in screening. This represents the single largest source of inefficiency within the screening programme, followed by missed findings and inconsistent participation. Given the improved outcomes associated with screen-detected cancers, there is a strong public health mandate to encourage participation.

## Introduction

Colorectal cancer (CRC, also referred to as ‘bowel cancer’) is the second leading cause of death from cancer in Europe.^1^ Several large randomised controlled trials (RCTs) have shown that regular faecal immunochemical test (FIT) screening, between the ages of 45 and 80, can significantly reduce the mortality of the disease among people who complete the test.^2^ As a result, many European countries have implemented FIT-based screening programmes for the early detection of CRC.^3^

England introduced its national bowel cancer screening programme in 2006. For the first four years, guaiac faecal occult blood test screening (gFOBt) was offered to men and women aged 60–69 years. In 2010, the programme was extended to include men and women up to the age of 74. Then, in 2019, the programme switched to biennial FIT-based screening for CRC, following the success of a national pilot, which demonstrated significant improvements in uptake (a one-off flexible sigmoidoscopy was also available for adults aged 55–59 from 2013 onwards; however, this was decommissioned in January 2021).^4^

Despite the availability of a national screening programme, which is free at the point of delivery, most CRCs in England are diagnosed outside of the screening programme. Indeed, a recent review of sex-related differences in routes to diagnosis found that only 8.1% and 5.1% of CRCs diagnosed in men and women, respectively, are diagnosed through screening.^5^

Why so many people are diagnosed with CRC outside of screening is not entirely clear. Indeed, ∼40% of people who are diagnosed with CRC are diagnosed at an age when they are eligible for screening (i.e. 60–74 years of age),^6^ so one might expect the proportion diagnosed through screening to be much greater. Possible explanations for the low diagnostic rate of screening include suboptimal use of screening among the eligible population (one study found that, while 70% of invitees participate at least once over three screening rounds, only 44% participate in all three rounds),^7^ false negative test results (at present, the programme uses a threshold of 120 μg/g to select individuals for colonoscopy; however, the cancer detection rate is almost doubled when a threshold of 20 μg/g is used)^4^ and non-attendance at follow-up colonoscopy (approximately 15% of adults who receive an abnormal FIT screening result do not attend a diagnostic investigation).^4^

To date, no studies have quantified the reason why so many individuals are diagnosed outside of screening, nor how route to diagnosis influences CRC outcomes in screening-eligible adults. The aim of this study, therefore, was to determine the extent to which diagnoses made outside the screening programme are due to non-participation in screening, false negative test results, and non-attendance at diagnostic investigation (i.e. colonoscopy), and how, if at all, these pathways influence stage at diagnosis.

## Patients / methods

### Study design

This study used a retrospective design to analyse data on 1011 adults diagnosed with CRC.

### Population

The population of interest was all men and women who were diagnosed with CRC at St Mark's Hospital (Harrow, UK), during a four year period (1st January 2017 – 31st December 2020).

### Setting

St Mark's Hospital is located within the Northwest London Borough of Harrow. It is responsible for screening the London boroughs of Brent, Harrow and North Ealing. Compared with the rest of England, these boroughs are more ethnically diverse (86%, 36% and 42% of individuals living in England, Harrow and Brent identify as White British, respectively), and Brent is more socioeconomically deprived (43% of people living in Brent do not own a car, for example, compared to 24% of people living in Harrow and 26% of people living in England).^8^

### Primary outcome measures

Data on the screening history and results of each individual were extracted from the bowel cancer screening system: an electronic system that provides up-to-date information about a person's bowel cancer screening status. Specifically, data on whether individuals had ‘never participated in bowel cancer screening’ and ‘participated in the most recent round of bowel cancer screening, prior to their diagnosis’, were extracted from the bowel cancer screening system, as well as whether the result was ‘positive’ (3 or more positive gFOBt samples [of 6], a FIT of >120 µg/g, or detection of adenomas / cancer during a flexible sigmoidoscopy), ‘weak positive’ (1 or 2 positive gFOBt samples [of 6]), or ‘normal’ (0 positive gFOBt samples [of 6], a FIT of <120 μg/g, or no adenomas / cancer during a flexible sigmoidoscopy). Where participants had a ‘positive’ result, additional data were extracted regarding whether they attended, or did not attend, a diagnostic investigation, such as colonoscopy.

### Secondary outcome measures

Data on several additional variables of interest were also extracted from the bowel cancer screening system, including sex (‘male’, ‘female’), age at diagnosis (measured continuously, in years), Ethnicity (‘White British’, ‘South Asian’, ‘Any other Asian ethnicity’, ‘Any Black Ethnicity’, ‘Any other White Ethnicity’, ‘Mixed/Other’), Region (‘Brent’, ‘Harrow’, ‘North Ealing’), area-level deprivation (measured using the ‘Index of Multiple Deprivation’ [IMD], which is the government's official measure for area-level deprivation, and uses census data to create a scale ranging from 0 to 80),^9^ Lymph node involvement at diagnosis (yes, no), Metastatic disease at diagnosis (yes, no) and the source by which individuals were diagnosed / referred for colonoscopy (screening, primary care, accident and emergency [A&E], other).

### Analysis

Descriptive statistics (i.e. frequencies and percentages) were used to describe the proportion of individuals who were eligible for screening, the demographic characteristics of those individuals, their previous screening participation, results and colonoscopy attendance, and the route by which they were diagnosed with CRC. Descriptive statistics were also used to report the involvement of lymph nodes and other organs at diagnosis.

Univariate and multivariate logistic regression were used to identify predictors of being diagnosed through screening (within the screening eligible sample) and having lymph node involvement and metastasis at diagnosis.

The threshold for statistical significance was 0.05; the data were analysed using SPSS (version 27.0).

### Missing data

Cases with missing data were excluded from the analyses.

### Ethics

Ethical approval was not required for this study, as it was considered ‘service evaluation’, as opposed to ‘research’, by the National Health Service (NHS) Health Research Authority.

## Results

### Sample characteristics

In total, 1011 patients were diagnosed with CRC at St Mark's Hospital between January 2017 and December 2020. Of these, 446 (44.1%) were eligible for screening at the time of diagnosis, the majority of whom were male (275/446, 61.7%) and of White British / Irish (174/446, 39.0%) or South Asian (94/446, 21.1%) ethnicity. The mean age and IMD score of screening eligible adults was 66.23 years (SD = 9.83) and 21.33 (SD = 9.83), respectively (Table 1).

**Table 1.** Sample characteristics of screening eligible adults diagnosed with CRC at St Mark's Hospital between January 2017 and December 2020.

Sex		N	%
Female		171	38.3
Male		275	61.7
Missing			
Age		Mean	SD
Age (years)		66.23	5.49
Ethnicity*		N	%
White British / Irish		174	39.0
South Asian		94	21.1
Any Other Asian Background		34	7.6
Any Black Background		39	8.7
Mixed / Other		17	3.8
Any Other White Background		30	6.7
Area-level deprivation (IMD Score)**		Mean	SD
IMD Score (0–80, least to most deprived)		21.33	9.83
Region		N	%
Brent		167	37.4
Harrow		151	33.9
North Ealing		128	28.7

*Data were missing for 58 (13.0%) patients.

** Data were missing for 23 (5.2%) patients.

IMD = Index of Multiple Deprivation.

### Screening participation and route to diagnosis

**Figure 1.**
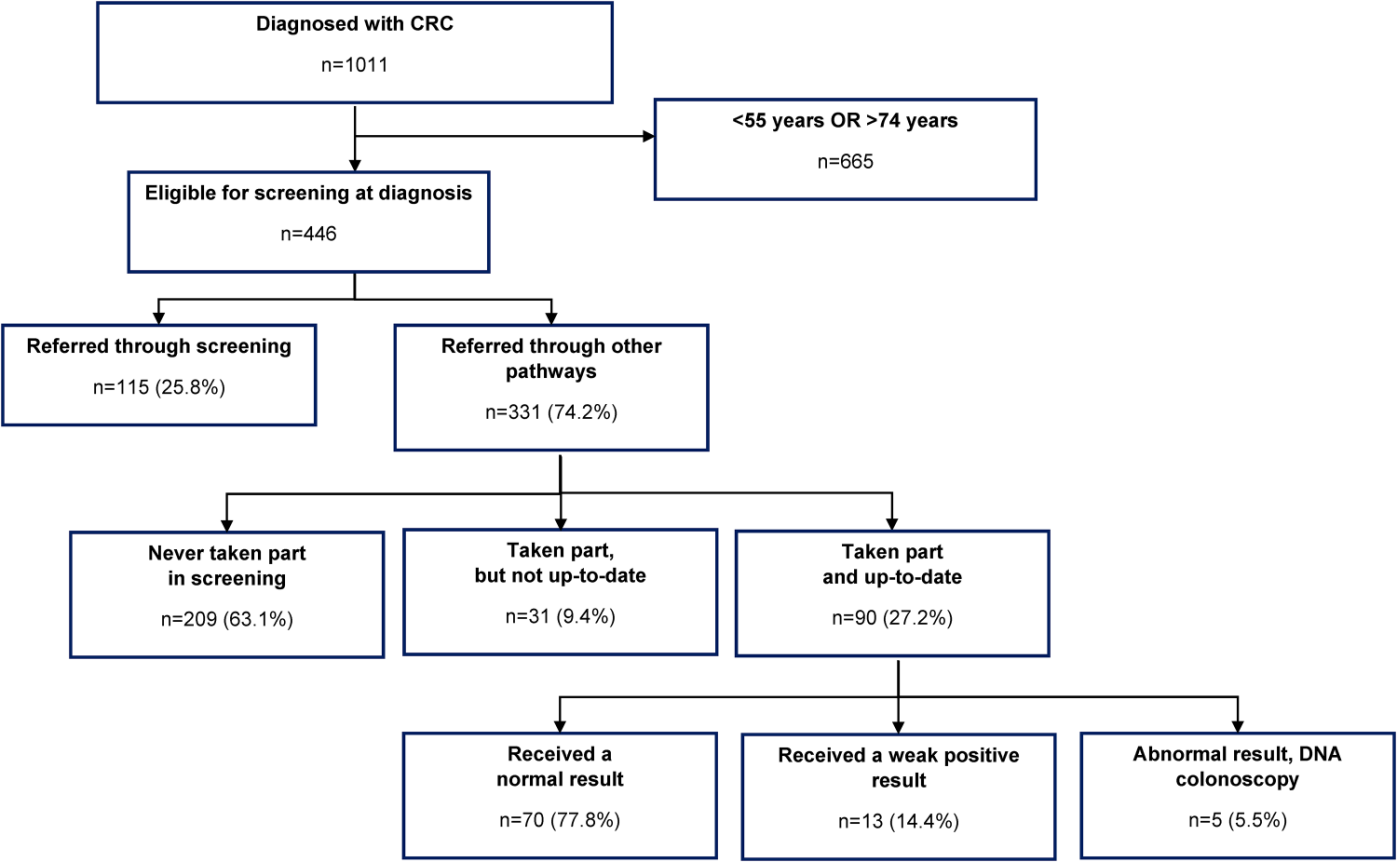
Clinical audit of men and women diagnosed with CRC at St Mark's Hospital: 2017–2020.

115/446 (25.8%) who were eligible for screening at the time of diagnosis were referred for colonoscopy through screening and 331/446 (74.2%) were referred for colonoscopy through non-screening pathways (217/331 [65.6%] through Primary Care, 58/331 [17.5%] through “other” pathways and 56/331 [16.9%] through A&E) (see Figure 1).

Among those who were referred for colonoscopy through non-screening pathways, 63.1% (209/331) had never taken part in screening, 9.4% (31/331) had previously taken part but were not up to date, and 27.2% (90/331) had taken part and were up to date (data were missing for 1/331 [0.3%]). Of those who had taken part and were up-to-date, 70/90 (77.8%) received a normal result for their most recent screening test, 13/90 (14.4%) received a weak positive result, and 5/90 (5.5%) received an abnormal test result and either did not attend the pre-colonoscopy assessment (3/90; 3.3%) of the colonoscopy (2/90; 2.2%) (see Figure 1).

### Predictors of route to diagnosis

In the multivariate analysis (Table 2), Asian individuals of a non-South Asian background (e.g. Nepalese) were significantly more likely to be diagnosed through screening than White British adults (47.1% vs. 27.0%; adjusted odds ratio [aOR]: 2.81, 95%CIs: 1.25, 6.29; *p*: 0.012), while individuals living in North Ealing were less likely to be diagnosed through screening than individuals living in Brent (16.4% vs. 35.5%; aOR: 0.36, 95%CIs: 0.19, 0.68; *p* = 0.002).

**Table 2.** Predictors of being diagnosed through screening.

		Diagnosed through screening n (%)	Univariate analysis OR (95%CIs)	Multivariate analysis (n = 379) aOR (95%CIs)
Sex				
	Female	52 (30.4)	1.00	1.00
	Male	63 (22.9)	0.68 (0.44, 1.05)	0.78 (0.48, 1.28)
Age				
	Age (years)	—	0.99 (0.95, 1.03)	0.98 (0.93, 1.02)
Ethnicity				
	White British / Irish	47 (27.0)	1.00	1.00
	South Asian	16 (17.0)	0.55 (0.29, 1.04)	0.66 (0.34, 1.28)
	Any Other Asian Background	16 (47.1)	2.40 (1.13, 5.10)*	2.81 (1.25, 6.29)*
	Any Black Background	13 (33.3)	1.35 (0.64, 2.85)	1.28 (0.57, 2.87)
	Mixed / Other	5 (29.4)	1.13 (0.38, 3.37)	1.26 (0.41, 3.87)
	Any Other White Background	10 (33.3)	1.35 (0.59, 3.10)	1.72 (0.70, 4.23)
Area-level deprivation (IMD Score)				
	IMD Score (0–80, least to most deprived)	—	1.01 (0.99, 1.03)	1.01 (0.98, 1.04)
Region				
	Brent	59 (35.5)	1.00	1.00
	Harrow	35 (23.2)	0.55 (0.34, 0.91)*	0.56 (0.31, 1.01)
	North Ealing	21 (16.4)	0.36 (0.20, 0.63)***	0.36 (0.19, 0.68)**

IMD = Index of Multiple Deprivation.

Bold-type indicates significance: * = *P* < 0.05; ** = *P* < 0.01; *** = *P* < 0.001.

### Predictors of lymph node involvement in the screening eligible population

In the multivariate analysis (Table 3), being referred for colonoscopy through general practice was associated with increased odds of lymph node involvement at diagnosis, compared with being referred for colonoscopy through screening (64.7% vs. 45.0%; aOR: 2.33, 95%CIs: 1.36, 4.01; *p*: 0.002). Similarly, having never participated in screening was associated with increased odds of lymph node involvement, compared with those who were diagnosed through screening (63.9% vs. 45.0%; aOR: 2.22, 95%CIs: 1.28, 3.86; *p*: 0.005, respectively).

**Table 3.** Predictors of lymph node and organ involvement at diagnosis.

		1 or more lymph nodes involved at diagnosis n (%)	Univariate analysis OR (95%CIs)	Multivariate analysis aOR (95%CIs)	Organ involvement at diagnosis n (%)	Univariate analysis OR (95%CIs)	Multivariate analysis aOR (95%CIs)
Sex							
	Female	78 (53.4)	1.00	1.00	24 (17.1)	1.00	1.00
	Male	148 (59.7)	1.29 (0.85, 1.95)	1.40 (0.87, 2.25)	45 (19.3)	1.16 (0.67, 2.00)	1.35 (0.71, 2.57)
Age							
	Age (years)	—	1.00 (0.96, 1.03)	1.01 (0.97, 1.05)	—	0.98 (0.94, 1.03)	0.97 (0.92, 1.03)
Ethnicity							
	White British / Irish	87 (57.6)	1.00	1.00	28 (19.4)	1.00	1.00
	South Asian	47 (56.6)	0.96 (0.56, 1.65)	0.95 (0.53, 1.69)	14 (18.9)	0.97 (0.47, 1.97)	0.77 (0.36, 1.66)
	Any Other Asian Background	21 (65.6)	1.40 (0.63, 3.12)	1.93 (0.81, 4.63)	5 (16.7)	0.83 (0.29, 2.36)	0.81 (0.26, 2.49)
	Any Black Background	17 (53.1)	0.83 (0.39, 1.79)	1.08 (0.46, 2.55)	4 (12.1)	0.57 (0.19, 1.76)	0.57 (0.17, 1.87)
	Mixed / Other	9 (52.9)	0.83 (0.30, 2.26)	0.91 (0.32, 2.58)	0 (0)	0.00 (0.00, 0.00)	0 (0)
	Any Other White Background	14 (50.0)	0.74 (0.33, 1.65)	1.06 (0.43, 2.62)	7 (25.9)	1.45 (0.56, 3.77)	1.14 (0.39, 3.40)
Area-level deprivation (IMD Score)							
	IMD Score (0–80, least to most deprived)	—	1.00 (0.98, 1.02)	1.00 (0.98, 1.03)	—	0.97 (0.95, 1.00)	0.96 (0.93, 1.00)*
Region							
	Brent	70 (50.0)	1.00	1.00	23 (16.8)	1.00	1.00
	Harrow	82 (59.4)	1.46 (0.91, 2.35)	1.68 (0.93, 3.05)	22 (17.5)	1.05 (0.55, 1.99)	0.72 (0.32, 1.59)
	North Ealing	75 (63.8)	1.76 (1.07, 2.91)*	1.71 (0.94, 3.08)	24 (21.8)	1.38 (0.73, 2.62)	1.39 (0.65, 2.95)
Source of referral							
	Screening	49 (45.0)	1.00	1.00	10 (9.8)	1.00	1.00
	GP Referral	130 (64.7)	2.24 (1.39, 3.61)***	2.36 (1.36, 4.01)**	33 (17.8)	2.08 (0.98, 4.42)	2.41 (1.01, 5.72)*
	A&E	22 (56.4)	1.59 (0.76, 3.31)	1.24 (0.52, 2.96)	12 (34.3)	5.01 (1.93, 13.01)***	4.39 (1.38, 13.98)*
	Other	25 (55.6)	1.53 (0.76, 3.08)	1.45 (0.67, 3.12)	14 (29.8)	4.07 (1.65, 10.05)**	4.09 (1.45, 11.56)**
Screening participation							
	Diagnosed through screening	49 (45.0)	1.00	1.00	10 (9.4)	1.00	1.00
	Participated and up-to-date	48 (60.0)	1.84 (1.02, 3.3)*	1.66 (0.84, 3.3)	15 (20.5)	2.48 (1.05, 5.89)*	2.96 (1.07, 8.24)*
	Participated, but not up-to-date	14 (58.3)	1.71 (0.70, 4.2)	1.91 (0.66, 5.57)	9 (39.1)	6.17 (2.14, 17.83)***	7.78 (2.26, 26.80)***
	Never participated in screening	115 (63.9)	2.17 (1.33, 3.52)**	2.22 (1.28, 3.86)	35 (20.6)	2.49 (1.18, 5.27)*	2.50 (1.05, 5.96)*

IMD = Index of Multiple Deprivation.

Bold-type indicates significance: * = *P* < 0.05; ** = *P* < 0.01; *** = *P* < 0.001.

### Predictors of metastasis in the screening eligible population

In the multivariate analysis (Table 3), being referred for colonoscopy through General Practice, A&E or Other routes was associated with increased odds of metastasis at diagnosis, compared with being referred for colonoscopy through screening (17.8% vs. 9.8%; aOR: 2.41, 95%CIs: 1.01, 5.72; *p*: 0.046; 34.3% vs. 9.8%; aOR: 4.39, 95%CIs: 1.38, 13.98; *p*: 0.012; 29.8% vs. 9.8%; aOR: 4.09, 95%CIs: 1.45, 11.56; *p*: 0.008, respectively). Similarly, having never participated in, not being up-to-date with, and being up-to-date with (but not referred for colonoscopy through) screening were all associated with increased odds of metastatic disease, compared with those who were diagnosed through screening (20.5% vs. 9.4%; aOR: 2.96, 95%CIs: 1.07, 8.24; *p*: 0.037; 39.1% vs. 9.4%; aOR: 7.78, 95%CIs: 2.26, 26.80; *p*: 0.001 and 20.6% vs. 9.4%; aOR: 1.05, 95%CIs: 1.05, 5.96; *p*: 0.039, respectively).

## Discussion

### Summary of main findings

This study found that only one in four CRCs diagnosed in screening eligible adults are diagnosed through the screening programme. In addition, this study found that most CRCs diagnosed outside the screening programme (six in ten) are diagnosed in people who have never taken part in bowel cancer screening, and that a considerable proportion (three in ten) are diagnosed in people who were up-to-date with their bowel cancer screening, but had received a normal or weak positive result that did not require further investigation (a small proportion were also diagnosed in those who received an abnormal result, but did not attend further investigation when invited).

This study also found that screening eligible adults who were diagnosed through the screening programme were less likely to have lymph node involvement at diagnosis, compared with those who were referred for colonoscopy through primary care (no differences were observed for those diagnosed through A&E or other pathways, although this may have been due to lower numbers [and thereby lower statistical power]). Patients diagnosed through A&E or other pathways were, however, more likely to have metastasis than those diagnosed through screening, as were those diagnosed through primary care.

Finally, this study found that individuals who were up-to-date with screening (but received a normal / weak positive result) or had never participated in screening were more likely to have lymph node involvement at diagnosis, compared with those who were diagnosed through screening, while those who had previously participated in screening but were not up-to-date were no more or less likely to have lymph node involvement (as above, this is likely due to small numbers and, thereby, reduced statistical power). Patients who had previously participated in screening but were not up-to-date were, however, more likely to have metastatic disease at diagnosis than those diagnosed through screening, as were those who had never taken part in screening, or had taken part and were up-to-date.

### Comparisons with the previous literature

The results of this study contrast with those of a larger study, conducted by White et al. (2018), which found that 8.1% of all CRCs diagnosed in men and 5.1% of all CRCs diagnosed in women are diagnosed through screening (present study: 11.2% [65/580] in men and 13.2% [57/431] in women). The reason for this discrepancy is not entirely clear; however, one possible explanation is that White et al. analysed data on people diagnosed with CRC between 2006 and 2013, before the age extension to 74 year olds was fully rolled-out (2014), and before the implementation of once-only flexible sigmoidoscopy screening for 55 year olds (also referred to as ‘bowel scope screening’), which started in 2013 and was decommissioned in 2021.^10^ Another possible explanation, or joint explanation, for the higher proportion diagnosed through screening is that White et al.'s study took place before the implementation of FIT, which began in 2019, and has higher uptake and sensitivity than gFOBt.^4,11^

The results of this study are, however, consistent with other studies examining disease progression by diagnostic pathway. For example, an analysis comparing the clinical outcomes of screen-detected cancers and stage-matched cancers found that more screen-detected cancers were diagnosed at Dukes’ Stage A, compared with interval cancers (40% [125/316] vs. 19% [36/187]).^12^

The finding that individuals from ‘Any other Asian background’ were more likely to participate in bowel cancer screening than their White British counterparts was unexpected. The authors believe these differences might be due to the Gurkha population, who are, anecdotally, highly compliant with screening (and form a large part of the local community). The finding that individuals from North Ealing were less likely to be diagnosed through screening than individuals from Brent, however, was expected and is likely due to lower screening participation in the region.^13^

### Strengths and limitations

This study has several strengths. First, it used objective measures of screening participation and history, as opposed to self-reported measures, improving the reliability of the findings. Second, it used data on an ethnically diverse sample, improving the generalisability of the results. Finally, there was a wide range of co-variates, including age, sex, area-level deprivation, screening history and route to diagnosis, reducing the effect of confounding data in the final models.

This study also has several limitations, First, it was restricted to a single centre in London, and so does not represent the general population of England. Second, the sample size was relatively small, meaning that the study may have been underpowered to detect modest differences between subgroups. Finally, as the study was restricted to data stored on the bowel cancer screening system, it was not possible to include other co-variates which may have been important / of interest (e.g. co-morbidities).

### Implications for policy and future research

This study has several implications for policy and research. First, the findings of this study reiterate the importance of health promotion campaigns encouraging regular participation in screening (not only did this study find that people who were diagnosed outside of the screening programme were more likely to have metastatic disease at diagnosis, but that this was true even of those who had taken part in screening, but were not up-to-date). Second, as the findings of this study were limited to a single centre in London, there is now a need to verify the findings with larger, national, datasets. Third, this study used data that were predominantly collected when gFOBt was the primary screening test, as opposed to FIT, and so repeat studies will be needed to see how this affects the proportion detected through screening in the future (given the analytical superiority of FIT, it is possible that some issues, such as ‘false negatives results’ and ‘the proportion of cancers missed among individuals who are up-to-date with screening’, may be overrepresented).

## Conclusions

This study demonstrates that the majority of screening eligible patients who are diagnosed with CRC are diagnosed outside of the screening programme. In addition, this study demonstrates that individuals diagnosed outside of the screening programme, including those who have previously taken part in screening but are not up-to-date, are more likely to be diagnosed with metastatic disease. The results of this study, therefore, reinforce the importance of regular participation in screening and the need for public health strategies to promote uptake among previous non-responders.
